# In Vivo Penetrating Microelectrodes for Brain Electrophysiology

**DOI:** 10.3390/s22239085

**Published:** 2022-11-23

**Authors:** Alexander Erofeev, Ivan Antifeev, Anastasia Bolshakova, Ilya Bezprozvanny, Olga Vlasova

**Affiliations:** 1 Laboratory of Molecular Neurodegeneration, Graduate School of Biomedical Systems and Technologies, Institute of Biomedical Systems and Biotechnology, Peter the Great St. Petersburg Polytechnic University, 195251 Saint Petersburg, Russia; 2 Laboratory of Methods and Instruments for Genetic and Immunoassay Analysis, Institute for Analytical Instrumentation of the Russian Academy of Sciences, 198095 Saint Petersburg, Russia; 3 Department of Physiology, University of Texas Southwestern Medical Center at Dallas, Dallas, TX 75390, USA

**Keywords:** neural interface, in vivo, brain, electrophysiology, neuronal activity

## Abstract

In recent decades, microelectrodes have been widely used in neuroscience to understand the mechanisms behind brain functions, as well as the relationship between neural activity and behavior, perception and cognition. However, the recording of neuronal activity over a long period of time is limited for various reasons. In this review, we briefly consider the types of penetrating chronic microelectrodes, as well as the conductive and insulating materials for microelectrode manufacturing. Additionally, we consider the effects of penetrating microelectrode implantation on brain tissue. In conclusion, we review recent advances in the field of in vivo microelectrodes.

## 1. Introduction

Electrophysiology has become a powerful technique for understanding the functioning of the brain, making a significant contribution to neuroscience [1,2,3]. Neurons in the brain are connected in a complex network, exchanging information with each other through action potentials (AP) [4]. This electrical activity (neural activity) is involved in brain functions such as behavior, perception and cognition. Historically, the most widely used instrument for recording neuronal activity has been the extracellular microelectrode [5]. Microelectrodes are used not only for recording neuronal activity, but also for electrical, optical and chemical neuromodulation [6,7,8,9]. To date, microelectrodes and neuroengineering approaches have benefited a large number of patients. For example, these include the partial restoration of vision with an artificial retina [10] and the restoration of the motor function of patients with paraplegia using a brain–computer interface [11,12]. Additionally, microelectrodes are used to study vesicular release on the cell by constant potential amperometry [13] or fast scanning cyclic voltammetry (FSCV) [14]. Both methods have proven critical to understanding brain chemistry and its role in behavioral neuroscience [15].

Different types of microelectrodes can be used for neuronal activity recording, such as in vitro and in vivo electrodes [16]. In vitro electrodes are most often microelectrode arrays (MEAs) [17,18,19], as well as glass microelectrodes for patch-clamp or long-term potentiation (LTP) registration [20,21]. In this review, we focus on penetrating microelectrodes for in vivo studies. We briefly consider their main types and materials, and we consider the effects of penetrating microelectrodes’ implantation on brain tissue. At the end of this review, we cover the current state of penetrating microelectrodes and multifunctional systems in neuroscience research.

## 2. Types of In Vivo Microelectrodes

**Figure 1:**
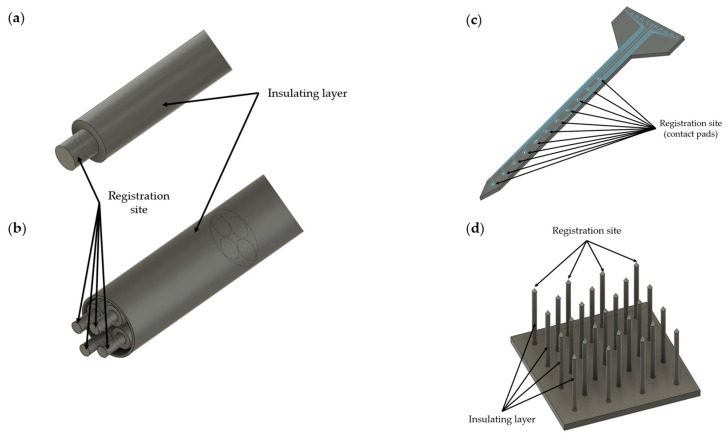
Schematic representation of different types of microelectrodes: (**a**) single-microwire neural electrode; (**b**) tetrode; (**c**) planar (Michigan) microelectrode; (**d**) Utah array.

In vivo electrodes can be divided into two main types: penetrating and nonpenetrating (for electroencephalography (EEG) and micro-electrocorticography (ECoG)). It should be noted that the electrodes for ECoG occupy an intermediate position because they are not directly injected into brain tissue, but their implantation requires surgery. The most interesting are penetrating electrodes (further microelectrodes) because they provide more information than nonpenetrating microelectrodes [16,22]. This type of microelectrode is implanted in a target area, usually the brain, and records neural activity: action potentials (APs) and local field potentials (LFPs) [16]. There are three main types of penetrating microelectrodes: microwire-type [23], Michigan-type [24] and Utah-type [25] (Figure 1).

Microwire-type electrodes are micron-sized metal wires covered with an insulating layer except for the ends of the wire. Microwire electrodes are divided into single-wire electrodes [26], tetrodes [27] and multiwire electrodes (microwire arrays) [23,28], and they have been used for both short-term and long-term neural recording in studies of rodents, primates and other laboratory animals [29]. The main advantage of these microelectrode types is the lower degree of tissue damage compared to Michigan-type [30,31] electrodes.

Michigan-type electrodes, in contrast to microwire electrodes, are less deformed during implantation due to the use of a rigid substrate, usually silicon. However, it should be noted that Michigan-type electrodes are more fragile than microwire electrodes. Electrodes of this type are manufactured with the microfabrication of microelectromechanical system (MEMS) [32,33] technology and are planar electrodes with a number from tens to thousands of contact pads (sites) located, most often, along the length of the microelectrode [16]. The main advantage of Michigan microelectrodes is the ability to simultaneously record neuronal signals at different levels of the brain tissue (multisite vertical recordings), while the disadvantage is a phenomenon of electrical crosstalk [34]. This phenomenon is associated with an increase in the coupling capacitance between electrodes and manifests itself more with an increase in the number of recording channels [34,35]. For neural microelectrodes, the recommended crosstalk level is about 1 percent of the recorded signal [34]. More details about the reasons for this phenomenon are described in references [34,35,36].

Utah-type microelectrodes, conversely, are designed in such a way that the contact pads (sites) are located on the tips of the needles (one needle, one recording channel) located vertically on a substrate made of silicon [37]. This type of microelectrode predominantly registers a large number (from hundreds to thousands) of neural signals at one level of the brain tissue (multisite horizontal recordings). Utah microelectrodes are FDA (U.S. Food and Drug Administration)-approved and applied in neuroscience research to understand the neural ensembles, as well as in brain–computer interfaces (BCIs) [38,39,40,41,42]. 

While Michigan- and Utah-type electrodes are state of the art, microwire-type electrodes (tungsten, stainless steel, platinum wire) are currently the most widely used microelectrodes in neuroscience [43,44]. The reason for this is the complexity of the fabrication process of the Utah- and Michigan-type microelectrodes compared to wire-type microelectrodes.

## 3. Materials for Microelectrodes

Since microelectrodes are intended for long-term implantation, biocompatible conductive (Table 1) and insulating materials are used in their manufacture. Table 1 presents various conductive materials, both biocompatible and toxic. For example, it is known that silver [45,46,47], copper [48], iron and cobalt [46] are toxic, in contrast to platinum and gold [49]. Biocompatible metals with good corrosion resistance [50] are used to implement the conductive properties of microelectrodes: gold [51], platinum [52,53,54,55,56,57,58], iridium [59], platinum–iridium [45], titanium [60] and tungsten [47]. These materials have good electrical conductivity and are well visualized by X-ray. The absorption of X-rays by the microelectrode metal makes it possible to visualize its state radiographically over time, which might be important in long-term implantation of the microelectrode [49]. It is worth noting that the increase in the atomic numbers of chemical elements relate to a decrease in the absorption of X-rays [61]. 

Alternatively, carbon-based materials are used as conductive materials [62]. Microelectrodes based on carbon nanotubes [62,63,64,65,66,67] and graphene [68,69,70,71] are being actively researched and manufactured. For example, the use of carbon fibers causes a lower immune response after implantation in the CNS compared to metal microelectrodes [72,73,74]. In addition, recently, conductive polymers [75,76,77] have been used as nontoxic materials, typical examples of which are poly-3,4-ethylenedioxythiophene (PEDOT) and its modifications [78,79,80,81,82,83,84,85,86,87,88], polypyrrole (PPy) [89,90,91,92] and polyaniline (PANi) [93,94,95].

Silicon is most often used as a rigid substrate in the manufacture of microelectrodes with MEMS technology and in the manufacture of needle microelectrodes [96,97,98,99,100]. Since silicon is a semiconductor material, it is covered with various insulating materials [101]. Some insulating materials include: varnish [102], glass [49,103], Teflon [104], silicon dioxide (SiO_2_) [105,106], silicon nitride (SiN) [105,107], silicon carbide [108,109], silicon dioxide with hafnium dioxide (HfO_2_/SiO_2_) bilayers [110], aluminum oxide (Al_2_O_3_) [111] and diamond-based coatings [101,112]. However, microelectrodes fabricated on a rigid substrate, such as silicon, cause inflammatory reactions, which further leads to microelectrode failure [31,75,113]. For this reason, microelectrodes on a flexible polymer substrate have appeared (Table 2), as well as rigid electrodes coated with a soft material such as hydrogel [114] or other insulating biocompatible materials. Among the polymers used are: flexible polyimide (Pi) [115,116,117,118,119], poly-para-xylylene (poly(chloro-p-xylylene)), in which the chlorine atom of one of the aromatic hydrogen atoms is substituted (Parylene-C) [120,121,122,123,124,125], and a negative photoresist based on epoxy resin (SU-8) [126,127,128,129,130], as well as a silicone elastomer, polydimethylsiloxane (poly(dimethyl siloxane), PDMS) [131,132,133,134], a prominent representative of which is Sylgard 184, which is often used in biological research [135]. To date, the organic polymer Parylene-C has become widespread [136]. This polymer is widely used in biomedical applications due to its resistance to harsh physical and chemical environments. In addition, Parylene-C is FDA-approved for implantation due to its high biocompatibility [137]. The sputtering of Parylene-C well covers the target structures; this polymer is widely used to isolate microelectrodes, with the exception of contact pads (sites), which register the electrical activity of the nervous tissue. More details about the structure and properties of Parylene-C can be found in the reviews by M. Golda-Cepa et al. [125] and by B.J. Kim et al. [138].

**Table 1:** Conductive materials for microelectrodes.

No.	Material	Atomic Number	Electrical Resistivity (20 °C, nΩ⋅m)	Electrical Conductivity (20 °C, 10^6^ S/m)	Thermal Conductivity, W/(m⋅K)	Thermal Expansion (25 °C, µm/(m⋅K))	Melting Point, °C	Biocompatibility	Description
1	Gold (*Au*)	79	22.14	44.2	318	14.2	1064	Nontoxic	Absence of gliosis [46]; widely used as a conductive material in the manufacture of microelectrodes.
2	Platinum (*Pt*)	78	105	9.3	71.6	8.8	1768	Nontoxic	Absence of gliosis [46]; platinum and platinum–iridium (*Pt-Ir*) widely used as conductive materials in the manufacture of microelectrodes.
3	Iridium (*Ir*)	77	47.1	21	147	6.4	2446	Nontoxic	Used as a conductive material in the manufacture of microelectrodes; has a high melting point and is rarely used for film microelectrodes [139]; iridium oxide (*IrO_2_*) has improved properties for electrical stimulation and registration of neuronal activity compared to iridium [140,141,142,143].
4	Tungsten (*W*)	74	52.8	8.9	173	4.5	3422	Nontoxic	Could be used as a conductive material in the manufacture of microelectrodes [46,144].
5	Tantalum (*Ta*)	73	131	7.7	57.5	6.3	3017	Nontoxic	Could be used as a conductive material in the manufacture of microelectrodes [145,146].
6	Silver (*Ag*)	47	15.87	62.1	429	18.9	961	Toxic	Silver (*Ag*) and silver chloride (*AgCl*) not recommended for microelectrode manufacturing [49]; may cause an allergic reaction [49]; formation of necrotic tissue [47,48].
7	Copper (*Cu*)	29	16.78	58.7	401	16.5	1084	Toxic	Formation of necrotic tissue [147,148]; not recommended for microelectrode manufacturing [49,147]; may cause an allergic reaction [49].
8	Nickel (*Ni*)	28	69.3	14.3	90.9	13.4	1455	Toxic	Not recommended for microelectrode manufacturing [147]; causes localized necrosis [147]; may cause an allergic reaction [49].
9	Iron (*Fe*)	26	96.1	10	80.4	11.8	1538	Toxic	Not recommended for microelectrode manufacturing [45,49].
10	Titanium (*Ti*)	22	420	2.4	21.9	8.6	1668	Nontoxic	Titanium and titanium nitride (*TiN*) could be used as conductive materials in the manufacture of microelectrodes [149,150,151]; titanium can oxidize, and for this reason, it is most often used as an adhesive layer [117,152,153].
11	Aluminum (*Al*)	13	26.5	36.9	237	23.1	660	Relatively harmless	Not recommended for microelectrode manufacturing [49,147].
12	Graphene	-	10	0.1	3500–5300	(−4)–(−3)	4236	Nontoxic	Could be used as a conductive material in the manufacture of transparent microelectrodes [71,154,155].
13	Stainless steel	-	690	1.45	15	14.4–17.3	1400–1530	Relatively harmless	Suitable for use as a conductive material in the manufacture of microelectrodes [48,156].
14	Nichrome	-	1100	0.909	13	14	1400	Nontoxic	Could be used as a conductive material in the manufacture of microelectrodes [48].

**Table 2:** Polymer substrates for microelectrodes.

No.	Name	Young’s Modulus (GPa)	Description
1	Flexible polyimide (Pi)	2.5 [116] 2.3–8.5 [139]	High thermal and chemical stability; polyimide thin films can be applied to microelectrode manufacturing; biocompatible [117]; it should be noted that certain types of polyimides readily absorb water, when polyimide is used as an electrical insulator, excessive water penetration can lead to corrosion and short circuits [157,158]; no FDA approval data.
2	Parylene-C	2.76 [120,139,159]	Organic hydrophobic polymer; high biocompatibility; well-shaped; sputtering ability [160,161,162]; FDA-approved.
3	SU-8	2.0 [127,128] 2.87–4.40 [163]	Well-shaped; used in microfluidics [164,165]; no FDA approval data.
4	PDMS	0.00132–0.00297 [132] 0.00036–0.00087 [139]	Used in microfluidic and nonpenetrating microelectrodes [166]; has high viscoelasticity, high gas permeability, a low dielectric constant and a low Young’s modulus, which can be changed by changing the curing temperature [167]; FDA-approved.

The thermal coefficients of an expansion conductor and insulator are vital to consider in situations where the insulating material needs to be applied to the microelectrode by heating [49]. This also requires the melting point of the insulation to be lower than the melting point of the metal composing the electrode [49], otherwise, the microelectrode insulation will be broken. A more in-depth study of the issue concerning the materials used to create microelectrodes for recording neuronal activity can be found in the reviews by S.M. Wellman et al. [168], A. Weltman et al. [139], G.H. Kim et al. [16] and L.A. Geddes and R. Roeder [49].

### Young’s Modulus

One of the important characteristics that should be considered in microelectrode manufacturing is Young’s modulus [168]. The Young modulus of brain tissue is less than ~10 kPa, while for silicon, this parameter is several orders of magnitude higher, ~190 GPa [16,139]. The mismatch between the stiffness of the brain tissue and the implanted microelectrode can lead to low and long-term inflammation due to pulsations (micromovements) of the brain tissue [139,169]. A. Sridharan et al. [170] showed that respiration can cause a force of 80–130 µN to act on the microelectrode by the surrounding brain tissue, while vascular forces can act on the microelectrode with a force of 14–25 µN [139]. Therefore, a greater mismatch between the Young modulus of the brain tissue and the microelectrode materials leads to extensive tissue damage around the implant. Moreover, the tethered microelectrode can shift its position by 2–25 µm during breathing and by 1–3 µm during heart contraction due to the forces acting on the implant [171]. A possible solution to the problem is the application of flexible materials for microelectrode manufacturing. J.K. Nguyen et al. [114] demonstrated that the application of a flexible microelectrode based on poly(vinyl acetate) with a Young modulus of ~10 MPa reduced the release of pro-inflammatory cytokines Iba1 and CD68 after implantation (2, 8 and 16 weeks) compared to a silicon microelectrode. After microelectrode implantation (16 weeks), NeuN (neuronal biomarker) staining revealed that neuronal death around the rigid silicon microelectrode was significantly higher compared to a flexible nanocomposite implant. Similar results were obtained in a study by J.P. Seymour et al. [172]. Flexible Parylene implants led to a neuronal loss of only 12–17% around the implantation site compared to rigid silicon microelectrodes (40%, significant neurodegeneration) four weeks after surgery. Alternatively, it is possible to use rigid silicon microelectrodes with various low-Young-modulus coatings [173,174], for example, hydrogel [78,114]. It is also worth noting that reducing the size of silicon microelectrodes to 3–5 µm significantly improves the parameters of the Young modulus [98,169].

## 4. Microelectrode Impedance

Small size microelectrodes (<15 µm of thickness for Michigan-type and <15 µm of diameter for wire- and needle-types) are most preferable for high-quality, long-term recording of neuronal activity [30,31] since their high spatial resolution allows them to record signals with a higher amplitude compared to larger microelectrodes [175] and they significantly reduce inflammatory processes at the site of the implantation. However, the microelectrode size (registration sites) reduction leads to an increase in impedance and negatively affects the signal-to-noise ratio (SNR) [176,177]. Low electrical impedance is an important characteristic that should be considered for neuronal activity recording because high impedance values, above 2–5 MΩ [86,178], can cause attenuation of the recorded signal [176,178]. The term “impedance” was introduced by Heaviside at the end of the 19th century to expand the concept of electrical resistance in AC circuits [179]. In this context, impedance is a measure of a system’s resistance to AC currents [180]. The impedance at the electrode–electrolyte interface (where the microelectrode surface interacts with the brain tissue) is a combined measure of the resistance to current flow through the electrode interface (resistance) and the ability to store charge at the interface (capacitive reactance) [181]. The charge in electronic systems is transported in the form of free electrons, while in biological tissues, it is transported in the form of charged ions [182]. For charge transfer between the microelectrode and the nervous tissue, an interface reaction is required. This can occur through two methods: (1) capacitive charging, in which the electrode–electrolyte double layer is charged and discharged, passing electrons between ions in the electrolyte and the metal conductor; (2) faradaic reactions, in which the electron transfer is facilitated by the oxidation or reduction of species at the interface [183]. It is worth noting that a double layer occurs at the electrode–electrolyte interface when a voltage is applied and behaves similarly to capacitors in the sense that it depends on the frequency. As the frequency increases, the reactance of the capacitor decreases exponentially. Due to the higher impedance inside the capacitor, at lower frequencies, current flows resistively. At higher frequencies, the impedance within the capacitor is greatly reduced and thus the current flows capacitively [184]. Thus, an accurate description of the impedance at the electrode–electrolyte interface is essential in designing microelectrodes and achieving a higher signal-to-noise ratio [185].

The impedance can be reduced by increasing the effective area and surface roughness of the microelectrode contact pad (site) [186]. Increasing the surface roughness leads to an impedance reduction without increasing the geometric size of the recording part of the microelectrode. Roughening can be achieved by chemical etching, electrolytic etching or mechanical abrasion (sandblasting). Additionally, a common way to increase the effective area consists of electroplating and deposition of the material [49]. These options are the most widely used. There are certain materials that are used for these options: platinum black (*Pt* black) [187], iridium oxide [151,188], Poly(3,4-ethylenedioxythiophene) (PEDOT) [79,87,88], Poly(3,4-ethylenedioxythiophene)-poly(styrenesulfonate) (PEDOT:PSS) [84] and hybrid materials based on iridium oxide and platinum black [189]. In addition, the impedance decrease is limited both by the area of the tip of the microelectrode recording part and by the impedance of the sputtering material. For this reason, another way of solving the problem of high impedance is becoming increasingly popular: amplifiers in the cascade of a microelectrode device, in addition to deposition of low-impedance materials [98,190,191,192,193,194,195]. Thus, Y. Kita et al. [98] described a needle electrode with a diameter of 3 μm and a length of 400 μm, produced using vapor–liquid–solid (VLS) silicon growth technology [196] on a modular conductive silicon block with a size of 1 × 1  mm^2^, placed on the microelectrode amplifier. The final device was a sandwich (STACK), where, on one side, there was a needle microelectrode 3 μm in diameter and 400 μm in length, and, on the other side, an amplifier module on a metal-oxide-semiconductor field-effect transistor (MOSFET AMP) with a size of 1 × 1 mm^2^ and a thickness of 525 μm. The connection between the microelectrode and the amplifier was achieved through a flexible intermediate layer based on polyimide with a thickness of 100 μm. As the authors noted, placing the amplifier on the microelectrode helps to reduce the noise between the electrode and the data acquisition system, in contrast to the configuration without an amplifier. This approach also solves the problems of attenuation of the recorded signal amplitude. The amplifier reduced the signal-to-noise ratio of a high-impedance 3 μm diameter microelectrode from 3.39 (10.6 dB) to 6.18 (15.8 dB).

Thus, the problem of increasing electrical impedance can be solved by modifying the surface of the contact pad and by setting up the amplifiers in the cascade of the microelectrode device. In the next part of this review, we consider the effects of penetrating microelectrode implantation on brain tissues.

## 5. The Effects of Microelectrode Implantation on Brain Tissue

The effects of penetrating microelectrode implantation on brain tissues according to the literature data [16,30,31,49,114,139,197,198,199,200] are as follows:

1. Disruption of the blood–brain barrier (BBB);
2. Tissue deformation;
3. Scarring of the brain tissue around the implant, i.e., gliosis [165,199,200,201];
4. Chronic inflammation after microelectrode implantation;
5. Neuronal cells loss.

All of the above effects except “tissue deformation” are associated with disruption of the BBB and inflammatory processes.

### 5.1. Disruption of the Blood–Brain Barrier and Inflammation

One of the most reported negative effects in the literature associated with the implantation of microelectrodes is inflammation [30,31,114,165,197,198,199,200,201,202,203,204]. For example, T. Saxena et al. [30] investigated the problem of inflammation and disruption of the BBB after the implantation of intracortical microelectrodes. To do this, the authors implanted planar (Michigan-type) microelectrodes with a thickness of 50 μm and microwire electrodes with a diameter of 50 μm into the brains of adult rats. After 16 weeks, they analyzed the microelectrode effectiveness with a quantitative analysis of BBB disturbance and subsequent infiltration of myeloid cells and neurotoxic factors. As a result, it was shown that planar microelectrodes showed significantly higher albumin accumulation compared to microwire electrodes. It should be noted that albumin extravasation is a generally recognized indicator of BBB impairment in various pathophysiological conditions [205]. In addition, the authors carried out the registration of action potentials caused by the stimulation of the whiskers of a laboratory animal for 16 weeks. The results of the electrophysiological recordings showed that Michigan-type microelectrodes ceased functioning after 10 days of implantation compared to microwire electrodes, which provided stable recording for up to 84 days. Thus, the importance of the microelectrode type selection and its size reduction (<50 µm) was noted.

A similar but more extended study was carried out by L. Karumbaiah and T. Saxena et al. [31]. This study evaluated the impact of various commercially available intracortical microelectrodes with histological, transcriptomic and electrophysiological analysis in acute (3 days) and chronic (12 weeks) periods. The acute period was characterized by the repair and inflammation of damaged tissue, and the influx of neutrophils and monocytes. The chronic period was characterized by long-term inflammation due to foreign material in the tissue, the persistent presence of lymphocytes and macrophages, and the formation of a fibrous capsule. The following microelectrodes were used: tethered (Michigan-type, 15 and 50 μm thick; microwire arrays), untethered (Michigan-type, 15 μm thick; arrays of floating microwire electrodes, 75 μm (floating microwire arrays, FMAA and FMAB)) and Utah microelectrodes. Tethered microelectrodes, implanted in the brain, are permanently attached to the skull, while untethered microelectrodes are not fixed to the skull and can move with the brain [206,207].

As a result, it was shown that smaller microelectrodes (15 µm of thickness for Michigan-type and 15 µm of diameter for wire-types) induced significantly less glial scarring and neuronal loss compared to larger electrodes (50 µm of thickness for Michigan-type and 50 µm of diameter for wire-types). At the same time, cylindrical microelectrodes (see Supplementary Materials) (microwire, 50 µm of diameter) caused significantly less glial scarring and disruption of the BBB compared to planar electrodes (Michigan-type, 50 µm of thickness) of the same size. It should also be noted that tethered Michigan-type microelectrodes (15 μm of thickness) caused significantly increased tissue scarring and damage to neurons compared to similar untethered microelectrodes (“floating” electrodes). This observation can be explained by the presence of micromovements at the site of the tethered microelectrode, which lead to increased reactive gliosis and rupture of brain microcapillaries. In addition, it was noted that microwire electrodes exhibited a significantly reduced expression of neurotoxic cytokine transcripts compared to other types of microelectrodes in chronic implantation. Increased expression of neurotoxic cytokines around the implanted microelectrode, in particular the TNF cytokine, can lead to the loss of neurons and microelectrode failure. TNF mRNA is secreted by activated microglia and stimulates glutamate production through autocrine activation of glutaminase, an enzyme that catalytically converts glutamine to glutamate, ultimately leading to excitotoxicity [31,208]. It has also been reported that the implantation of microelectrodes of all types leads to chronic activation of the pro-inflammatory cytokine IL1b. These data indicate the need for therapeutic intervention, e.g., IL1Ra receptor antagonists, to reduce chronic IL1b-mediated neurotoxicity.

Thus, the following can be noted:

1. The microelectrode type, size (thickness, diameter) and tethered or untethered form are important factors affecting both the lifetime of the microelectrode and the quality of the obtained data (change in impedance and signal-to-noise ratio).
2. Applying untethered microwire electrodes with a small size (<15 µm of thickness, diameter) could reduce BBB damage, glial scarring and neuronal loss, thereby potentially extending the lifetime of a long-term implanted microelectrode and improving the quality of the obtained data.
3. Coating microelectrodes with anti-inflammatory drugs or their additional injection can reduce chronic neurotoxicity mediated by pro-inflammatory cytokines.

**Figure 2:**
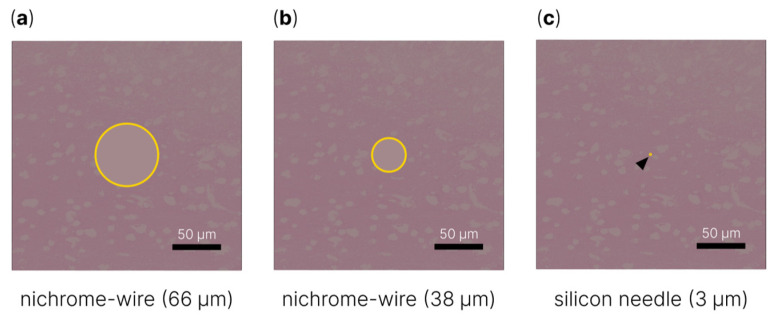
Schematic representation of tissue damage area after implantation of (**a**) 66 μm diameter nichrome wire, (**b**) 38 μm diameter nichrome wire and (**c**) 3 μm diameter silicon needle [99].

It is also worth noting the need to reduce the size of the microelectrode for long-term recording of neuronal activity [209]. The article by A. Fujishiro et al. [99] presented the results of immunohistochemical tissue analysis after the implantation of 66 and 23 µm nichrome wires and a 3 µm silicon microneedle 4 days after surgery. The data presented in this paper clearly show how little tissue damage area occurs when using a microelectrode with a diameter of 3 µm (Figure 2).

After several reports on the importance of microelectrode size reduction for chronic in vivo recording, several articles reported on microelectrodes <15 µm in diameter: with a tip diameter of 8.5 µm [73] (2012); with a tip diameter of 3 µm and a length of 210 µm [99] (2014); with a tip diameter of 5 µm and a length of 160 µm [97] (2016); with a tip diameter of 5 µm and a length of 400 µm [210] (2017); and with a tip diameter of 3 µm and a length of 400 µm [98] (2021). S. Yamagiwa et al. [210] demonstrated the fabrication of a microelectrode with a diameter of 5 µm and a length of 400 µm with VLS technology on a 1 × 1 mm^2^ modular conductive silicon block. After the VLS growth process, the microneedle was platinum (*Pt*)-plated with a titanium (*Ti*) adhesive layer and encapsulated with a biocompatible Parylene-C insulator, except for the microelectrode tip, which was coated with a low-impedance platinum black (*Pt* black) material due to its high impedance characteristics. The authors noted that due to the small size, the fabricated microelectrode smoothly penetrated into the brain of a laboratory animal without significant tissue deformation (~4.3 μm), while for a microelectrode with a diameter of ~80 μm, 20 times greater tissue deformation, ~94.2 μm, was observed. It should be noted that the VLS technology makes it possible to create longer microelectrodes (>400 µm) of the same diameter. However, in this case, the needle frame needs to be strengthened with a soluble silk film to reduce the probabilities of microelectrode deformation and failure [211].

### 5.2. Chronic Inflammation

As noted earlier, the brain tissue has micromovements (pulsations) that lead to long-term inflammation of the tissues around the implant and can lead to displacement of the microelectrode [170,171]. This problem can be solved both by the decrease in the material’s Young modulus and by application of an untethered (floating) microelectrode [212]. It was previously mentioned that implanted untethered microelectrodes cause less tissue scarring and neuronal damage [31]. K. Yamashita et al. demonstrated a “floating” needle microelectrode with a diameter of 5 μm and a length of 400 μm, manufactured with the VLS technology [100,169]. This microelectrode is called a floating microelectrode because it is connected by a flexible wire to a data connector and implanted in the mouse brain without fixation to the skull. Both a “floating” microelectrode and a microelectrode fixed on the skull were implanted into the visual cortex of mice for recording neuronal activity induced by light stimuli. After 7 days, the degree of tissue injury was assessed. As a result, after formalin perfusion fixation of the tissue, it was confirmed that the “floating” electrode led to less tissue damage area than a similar tethered needle microelectrode. 

## 6. Modern Advances in the Field of In Vivo Penetrating Microelectrodes for Brain Electrophysiology

The biggest advantage of implantable devices, particularly penetrating microelectrodes, for electrophysiological recording is the high temporal resolution of the biological signal registration in real time [16,213]. If a high-density recording of neural activity with a resolution of one neuron is added to this, we could have a powerful tool for understanding neural networks at the cellular level [214,215] and providing the most precise control in brain–machine interface applications [1,173]. For information about brain–machine interfaces, readers may refer to the reviews by A.B. Rapeaux et al. [216] and S. Saha et al. [217].

**Figure 3:**
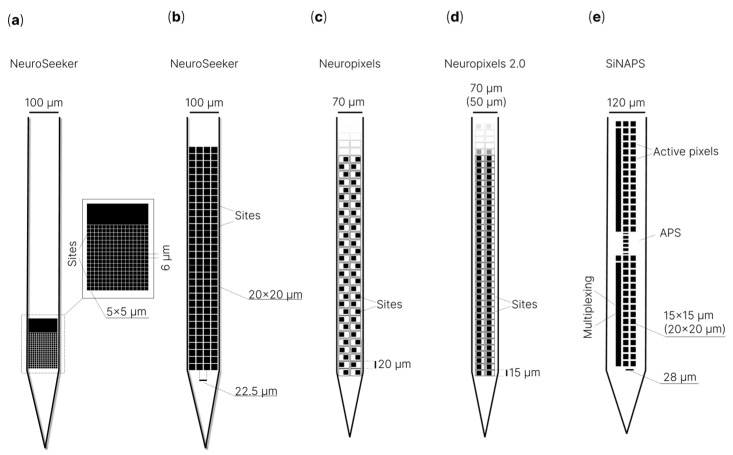
Schematic illustration of microelectrodes for high-density recording of neuronal activity: (**a**) 128 channel silicon probe (NeuroSeeker) [222]; (**b**) 255 channel silicon probe (NeuroSeeker) [223]; (**c**) NeuroPixels [213]; (**d**) NeuroPixels 2.0 [219]; (**e**) SiNAPS [226].

Based on Michigan-type electrodes, a number of microelectrodes for high-density recording of neuronal activity (Figure 3) have been implemented: a 3D (three-dimensional) silicon probe [218], NeuroPixels [213], NeuroPixels 2.0 [219], NeuroSeeker [220,221,222,223], Neurotassel [224], Neuralink [225], SiNAPS [226] and CMOS microwire array [23,227,228].

**Figure 4:**
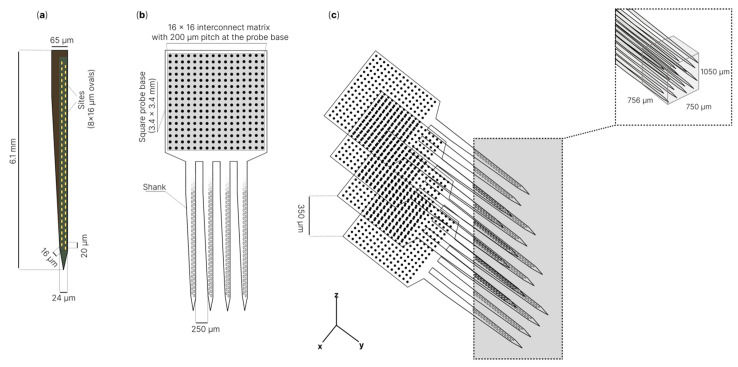
Schematic of three-dimensional neural probe for dense recordings: (**a**)—example of microelectrodes shank; (**b**)—256 channel microelectrode with 4 shanks, intershank spacing 250 μm. 256 electrodes connected to 16 × 16 interconnect matrix with 200 μm pitch at the probe base; (**c**)—three-dimensional electrode array with 1024 electrodes density per 0.6 mm^3^ [218].

G. Rios et al. [218] described three-dimensional electrode arrays (Figure 4) and a modular, scalable system for dense electrophysiology recordings. The authors presented minimally invasive shanks that were stacked into a three-dimensional array with over a thousand recording sites. Each neural probe was a silicon device with a square base (3.4 × 3.4 mm) 21 µm thick, including several shanks 6.1 mm long, 65 µm wide at the base and 24 μm near the tip. The total number of recording channels was 256 (oval-shaped microelectrode pads, 8 × 16 μm). Microelectrode pads were connected to a 16 × 16 interface matrix of circular pads (100 μm in diameter and edge-to-edge spacing) on a square base. All shanks were coated with Parylene on three sides, while the backside was made of silicon oxide. The developed microelectrode array was successfully tested; the authors recorded the electrophysiological activity from the hippocampus of awake mice with a fixed head using a 1024 electrode three-dimensional array. It is noted that the presented technology can be further improved by increasing the number of recording sites by an order of magnitude while maintaining a modular architecture and a small array size.

J.J. Jun et al. [213] described the NeuroPixels microelectrode, which has 960 low-impedance registration channels based on titanium nitride [150,229] (contact sites), arranged in a checkerboard pattern with dimensions of 12 × 12 µm and a distance between them of 20 µm from center to center on a single 10 mm long tile with a shank with a cross-section of 70 × 20 µm. Such a high density of recording channels was achieved by using a special 130 nm CMOS fabrication process [230]. At the same time, NeuroPixels uses 384 out of 960 registration channels simultaneously, programmatically switching between the rest of the channels. The authors noted that with the help of two NeuroPixels microelectrodes, it was possible to simultaneously register more than 700 signals from single neurons from five brain structures of an awake mouse. Stringer et al. [231] revealed that, with a NeuroPixels probe and two-photon calcium imaging, the population activity of the visual cortex reliably encodes an orthogonal fusion of sensory and multidimensional behavioral information.

Subsequently, N.A. Steinmetz et al. [219] introduced the second version of NeuroPixels, NeuroPixels 2.0. In this version of the microelectrode, the recording channels are vertically aligned instead of staggered to implement the motion correction algorithm, with an interelectrode distance of 15 µm instead of 20 µm, which ultimately makes it possible to have 1280 pads (site) instead of 960, as in the first version. The authors noted that two four-shank NeuroPixels 2.0 probes provide 10,240 recording channels in one implant. This NeuroPixels 2.0 variant may be a promising tool for large-scale recording of neuronal activity in the brain.

“NeuroSeeker” [220,221,232] is a similar project, which is also a microelectrode with low-impedance registration channels based on titanium nitride, made according to 130 nm CMOS technology. This type of microelectrode has a shank that is 8 mm long with a cross-section of 100 µm × 50 µm. “NeuroSeeker” has been presented in several versions: 128, 255 [221,232] and 1356 [220] registration channels. Registration sites are 20 × 20 μm in size with distances between the edges of the pads of 2.5 μm. The presence of densely spaced registration channels, coupled with the size of the microelectrode, makes it possible to simultaneously record local field potentials and action potentials in the sensory cortex, hippocampus and thalamus [220]. As noted earlier in this review, Michigan-type microelectrodes simultaneously record a large number of neuronal signals at different levels of the brain tissue (vertical multisite recording). However, this microelectrode type is useless for large-scale neuronal recording at one level of the brain tissue (horizontal multisite recording), which is important for researching brain functioning. To solve this problem, three-dimensional microelectrode arrays have been created, which combine the advantages of Michigan- and Utah-type microelectrodes. G. Rios et al. [218] proposed a three-dimensional array with more than a thousand channels for registration as mentioned before. A similar approach was taken by J.E. Chung et al. [233] when using polymer microelectrodes.

We should also mention the project that combines the best solutions from the NeuroPixels and NeuroSeeker projects, namely, SiNAPS [226]. G.N. Angotzi et al. presented a modular active-pixel sensor (APS) high-resolution CMOS-based microelectrode realized in a 0.18 μm CMOS technology, with an implantable single-shaft probe with a regular array of 512 electrode pixels with a pitch of 28 µm. The authors presented an APS CMOS probe technology for neural recording with readouts at 25 kHz/channel from up to 1024 electrode pixels. The presented SiNAPS (simultaneous neural active-pixel sensor) CMOS-based microelectrode permits simultaneous readouts from all electrode pixels in the array while minimizing the total *Si* area. The microelectrode successfully registered local field potentials and action potentials in the somatosensory cortex area of an anesthetized rat. As the authors noted, the modularity and characteristics of the SiNAPS technology could pave the way for a new generation of highly integrated implantable probes for different varieties of animal models and applications.

Another interesting project for high-density recording of neural activity with CMOS technology is the development of the Argo system [23,227,228]. The basic idea is to use a large number of microwire electrodes connected perpendicular to the plane of the CMOS amplifier array. The result is a device similar to the Utah array. The prototype Argo system has the ability to continuously record 65,536 channels at 32 kHz and 12-bit resolution. The performance of this microwire array was confirmed through the registration of LFPs in the auditory cortex of sheep [234].

**Figure 5:**
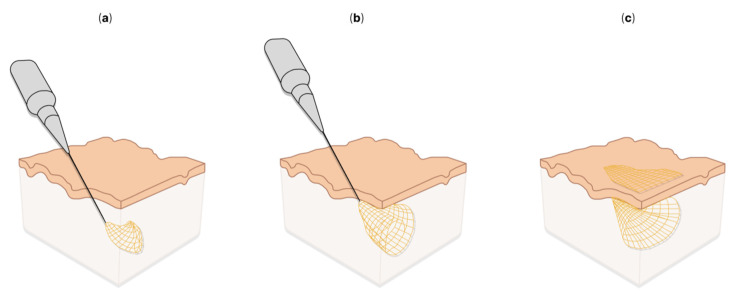
Syringe injection electronics: (**a**–**c**)—schematics of injectable electronics [245].

High-density silicon-based microelectrodes and their arrays [213,220,222,226,235,236,237,238,239,240] are powerful tools for simultaneously recording hundreds of neurons with high temporal and spatial resolutions. However, it is worth considering that Michigan-type microelectrodes lead to a long-term immune response that causes glial scarring, neuron loss surrounding the implant and failure of the recording channels [31,74,241,242,243]. These limitations hinder basic research in neuroscience as well as clinical applications. In this regard, it is worth noting the development of other interesting directions, such as flexible CMOS-compatible probes [244] or mesh microelectrodes, which are mainly used for ECoG in the form of film microelectrode arrays. J. Liu et al. [245] developed a flexible microelectrode mesh array with 16 recording channels, which, in a folded state, was introduced into the brain tissue through a needle with a diameter of 100 μm (Figure 5). The authors emphasized that this method is applicable for the delivery of flexible electronics through rigid organic shells to fill internal cavities, and co-injection with other materials. The application of mesh microelectrodes can reduce neuroinflammation after implantation [246].

Tian-Ming Fu et al. [247] also described a mesh microelectrode, but with the number of recording channels increased from 32 to 128. This microelectrode successfully recorded local field potentials and the activity of single neurons from several brain regions in awake mice for 4 months. As the authors noted, this development represents important progress towards the realization of ideal implantable microelectrodes.

Mesh microelectrodes were applied to endovascular brain–computer interfaces by the “Synchron” company. This company has performed a series of successful surgeries to implant mesh cylindrical microelectrodes (stent rods) into the blood vessels of the brain of a patient with amyotrophic lateral sclerosis [248,249]. Patients with an implanted stent rod may perform tasks such as texting and making online purchases.

Another interesting development is Neurotassel described by S. Guan et al. [224]. Neurotassel is an array of flexible, high-aspect-ratio microelectrode filaments that can be assembled into a thin implantable fiber through elastocapillary interactions. Neurotassel implantation causes minimal loss of neuronal cells in the brain and offers a new approach for stable neural activity recording and neuroprosthetics. In addition, Neurotassel can be easily scaled up to 1024 microelectrode filaments (3 × 1.5 µm^2^). It is also worth highlighting ultra-flexible electrode arrays made of nanomaterials with a cross-sectional area of less than 10 μm^2^, as described by X. Wei et al. [250].

Neuralink, the development of E. Musk [225], should also be mentioned, which is an array of flexible microelectrode “threads” with 3072 recording channels per array, distributed over 96 “threads”. The main substrate and dielectric used in these arrays of flexible microelectrodes is polyimide, which encapsulates the track as a thin gold film. To reduce the impedance of gold pads (sites), PEDOT:PSS and iridium oxide (IrO_2_) were used for various modifications of flexible microelectrode “threads”. To implant this array of small and flexible electrode “threads”, a neurosurgical robot was developed based on the work of T.L. Hanson et al. [251], which is capable of implanting six “threads” (192 recording channels in total) per minute. An important advantage of this approach is, as noted, the high accuracy (up to a micron), which avoids damage to the vascular network and, as a result, reduces tissue damage and neuroinflammation. Before implantation, Parylene-C is applied to the microelectrode array, forming a film on which the “threads” remain attached until the surgical robot pulls them out. Each “thread” ends with a 16 × 50 µm^2^ loop for threading the needle, i.e., the implantation process is similar to using a sewing machine. Testing of two modifications of flexible arrays with 1536 (simultaneous recording from 1344 channels) and 3072 channels (simultaneous recording from all channels) was successfully carried out on rats. As a result, both local field potentials and activity from individual neurons were recorded. As the author noted, Neuralink is a research platform for application in rodents and serves as a prototype for future human clinical implants. More details about automated implantation systems can be found in the review by D. Atkinson et al. [252]. It is worth noting that a Neuralink array of flexible microelectrodes has a depth of implantation no more than 2 mm, while for Michigan-type microelectrodes, this is more than 5 mm [218].

**Figure 6:**
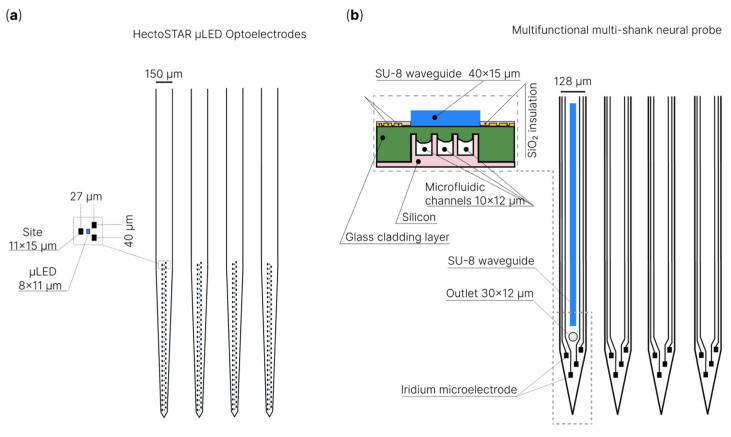
Schematic illustration of multifunctional systems that combine optical stimulation, microfluidic injections with electrophysiology: (**a**) HectoSTAR μLED optoelectrodes for in vivo optical electrophysiology [272]; (**b**) multifunctional multi-shank neural probe [44,275].

It is also worth highlighting multifunctional systems that combine optical stimulation (optogenetics, infrared stimulation, microfluidic injections) with electrophysiology [253,254,255,256,257,258,259,260,261,262,263,264,265,266,267,268,269,270,271]. For example, K. Kim et al. developed the HectoSTAR µLED optoelectrode (optrode) [272], which is a microelectrode combined with µLED light-emitting diodes (Figure 6a). The optoelectrode is made with miniSTAR [270] technology, and it has 256 registration channels and 128 LEDs distributed over four rods and covers a large volume with a cross-sectional area of 900 μm by 1300 μm. As the authors noted, the device makes it possible to simultaneously register neurons distributed inside a large volume and act selectively on them with a high (<40 μm) spatial resolution. J.W. Reddy et al. demonstrated a flexible double-sided microelectrode on a Parylene-C substrate combined with a 22 × 22 µm (445 nm, 200 µW at 2 mA) gallium nitride µLED for chronic use [273]. It was noted that the presented technology makes it possible to fabricate ultra-compact high-density optoelectronic neural sensors. In addition, the proposed technology will make it possible to achieve high productivity of the fabrication process for a large number of optoelectrodes. T.V.F. Abaya et al. introduced an optrode based on a Utah array for optogenetic and infrared neural stimulation [253,254]. Á.C. Horváth et al. presented a device with monolithically integrated optical, thermal and electrophysiological functions [274]. The device with high accuracy controls the spatial and temporal distribution of temperature in the deep tissues of the brain and simultaneously registers the neuronal activity of individual cells. The performance of this multifunctional microelectrode was successfully demonstrated in the rat neocortex and hippocampus by increasing or suppressing the firing rate of stimulated neurons using continuous infrared light. The authors noted that their device could be a promising device for detecting thermally evoked responses in deep brain tissues. Except for µLED, quantum dots are used to create optrodes; more details on this topic can be found in [266].

In addition to optogenetic stimulation, neuronal activity can be modulated through the delivery of various biological agents targeting channels or receptors via microfluidic channels. Michigan-type silicon probes are a classic example of a multifunctional device that registers neuronal activity and has a microfluidic channel [275,276,277]. In these microelectrodes, microfluidic channels are formed by deep reactive ion etching of the silicon substrate. Due to the presence of a microfluidic channel in the microelectrode, ictal patterns were successfully recorded by H.J. Lee et al. along with the simultaneous electrophysiological recording of neuronal activity during microinjection of baclofen (a drug that causes convulsions) [276]. H. Shin et al. proposed a microelectrode with multidrug delivery capability suitable for small animal experiments [44,275] (Figure 6b). The authors demonstrated the successful infusion of various chemicals (pilocarpine or tetrodotoxin, buffer solution and 4′,6-diamidino-2-phenylindole) locally into the mouse brain. As a result, the administration of neuromodulatory drugs led to an increase or decrease in neuronal activity, which was confirmed through simultaneous electrophysiological recording. In addition to rigid silicon microelectrodes, microfluidic channels have been implemented on flexible microelectrodes. A. Altuna et al. presented an SU-8-based microelectrode for neuronal recording and drug delivery [126]. It should be noted that microfluidic channels were predominantly integrated into Michigan-type microelectrodes due to their two-dimensional structure, while this was difficult for three-dimensional microelectrodes. However, a few studies on multifunctional needle-type arrays with microfluidic channels have been reported [278,279,280]. For example, Y.N. Kang et al. fabricated a microfluidic interconnect cable (µFIC) and integrated it into a three-dimensional flexible penetrating microelectrode array (FPMA), consisting of silicon microneedle electrodes on a flexible substrate [280]. The developed device successfully carried out the delivery of potassium chloride, simultaneously recording neuronal activity in vivo. Additionally, it is worth mentioning systems that combine electrophysiological registration, optical stimulation and the presence of microfluidic channels [281,282]. In these multifunctional systems, microfluidic channels are used to deliver viruses or to deliver drugs. B. Rubehn et al. presented a device that can conduct light and fluids to a target area of the brain and simultaneously record neural activity from the same part of the tissue [283]. To achieve this, they used microsystem technology to integrate an SU-8 waveguide and fluid channel into a polyimide electrode shaft. Shin et al. demonstrated a multi-shank MEMS microelectrode with an optical waveguide and microfluidic channels for drug delivery [44]. The performance of this microelectrode was demonstrated by confirming and modulating the functional connectivity between the hippocampal CA3 and CA1 regions in vivo. K. Sharma et al. also demonstrated a multifunctional device that combines a silicon-based microelectrode with an integrated microfluidic channel and an optical fiber in a compact package [269]. The device successfully recorded neuronal activity in the prefrontal cortex after photostimulation in a freely moving rat for 9 weeks after implantation. In addition, the authors injected the GABA antagonist bicuculline into the anesthetized rat brain and simultaneously recorded the electrophysiological response. The use of modern multifunctional devices provides higher spatial specificity and minimal damage to brain tissue.

Finally, we would like to briefly mention additive technologies, also known as 3D printing, which at the nanoscale [284,285] can be used for microelectrode fabrication. For example, M.S. Saleh et al. [286] presented a nanoparticle 3D printing approach to create in vivo electrodes with a high density of recording channels (2600 channels per cm^2^). The nanoscale 3D printing technology made it possible to fabricate configurable electrodes with different individual shank lengths and layouts, with low channels impedance for targeted and large-scale neural activity recordings [286]. T. Roy et al. [287], proposed the topology optimization algorithm [288,289] to create porous electrodes with a given structure without compromising its rigidity. High-precision 3D printing is a new area, but it already has good prospects for the future in the field of the fabrication of microelectrodes and biomedical devices. Nanoscale 3D printing provides inexpensive and rapid custom design prototyping without the constraints of traditional manufacturing [286]. More details about 3D printing for biomedical and electronic devices can be found in the reviews by L. Line et al. [290], by K. Muldoon et al. [291] and Y. Liu et al. [292].

Thus, new technologies and approaches significantly expand the possibilities in the research of real-time biological signal recording and brain mapping and the understanding of the neural networks associated with behavior, perception and cognition.

## 7. Conclusions

The development of technologies has led to the creation of new improved in vivo penetrating microelectrodes for the study of neural networks and brain functions. Conductive polymers, nanomaterials and sputtering insulating materials have been actively used in the microelectrode fabrication process with the development of technology. More and more attention is being paid to matching the mechanical properties of brain tissue and microelectrode materials. The development of microelectromechanical systems and micromachining technologies has reduced the size of electrodes. Microelectrodes have appeared with thousands of channels for recording neural activity. In addition, various designs of microelectrodes have been created, such as three-dimensional silicon probes and multifunctional systems combining optogenetic stimulation and microfluidic injection. Thanks to these advances, the sensitivity and selectivity of microelectrodes have been improved, as well as their lifetime increased due to improved biocompatibility and size reduction.

In this review, we provided the necessary information to comprehend the area of chronic recording of neuronal activity in brain tissues. We considered the main components of microelectrodes: conductive and insulating materials, and the main types of microelectrodes. In addition, attention was paid to Young’s modulus and microelectrode impedance, as well as the effect of penetrating microelectrodes on brain tissue. Finally, we reviewed examples of recent achievements in the field of in vivo microelectrodes.

Modern microelectrodes and multifunctional systems can be used in various applications, such as the diagnosis and treatment of diseases, prosthetics and the creation of brain–computer interfaces, leading to the next generation of neuroscience and medicine.

## Data Availability

This study did not report any data.

## Supplementary Materials

The following supporting information can be downloaded at: https://www.mdpi.com/article/10.3390/s22239085/s1.
