# Supplementary figures and images for: In Vivo Penetrating Microelectrodes for Brain Electrophysiology
Source: Sensors (Basel). 2022 Nov 23;22(23):9085. doi: 10.3390/s22239085 (PMC9735502; doi:10.3390/s22239085)

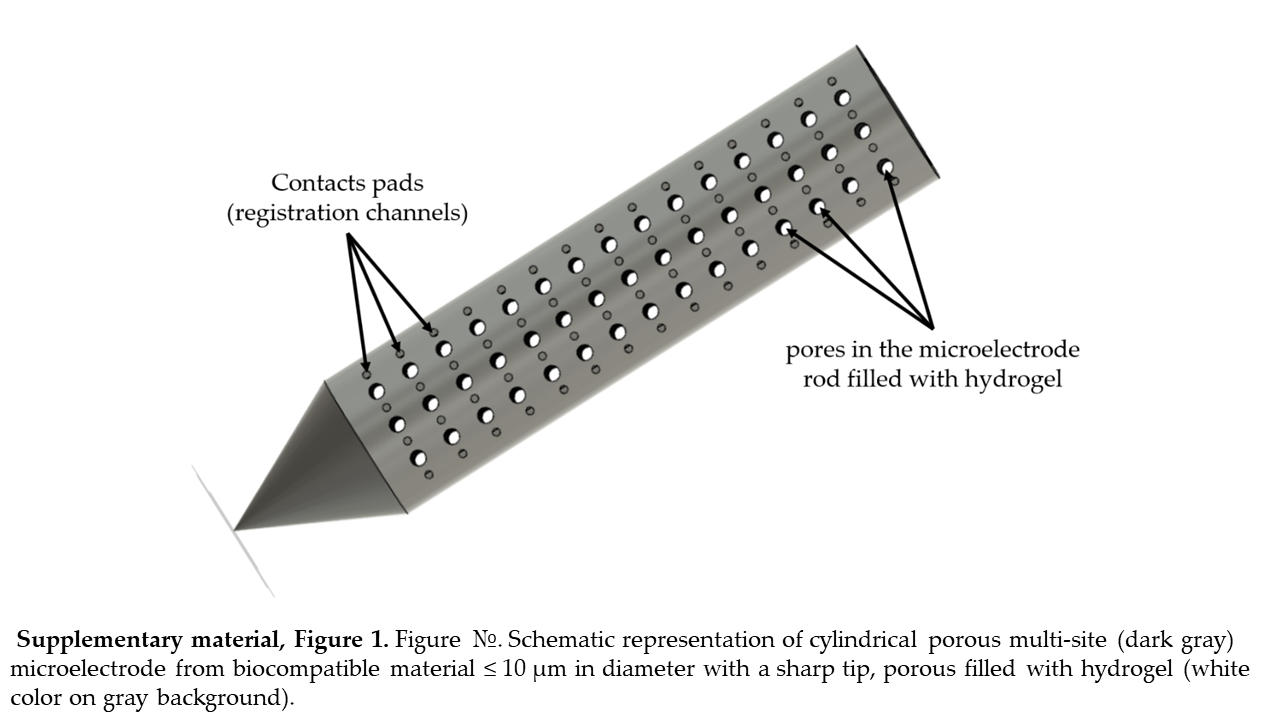

Supplement: Supplementary file 1 [file sensors-22-09085-s001.zip › Supplementary_material_figure_1.png]
